# In vitro α-amylase inhibitory effect of TLC isolates of *Aloe megalacantha* baker and *Aloe monticola* Reynolds

**DOI:** 10.1186/s12906-019-2622-5

**Published:** 2019-08-07

**Authors:** Gebretsadkan Hintsa Tekulu, Ephrem Mebrahtu Araya, Hayelom Gebrekirstos Mengesha

**Affiliations:** 1 Department of Pharmacognosy, School of Pharmacy, College of Health Sciences, Mekelle University, P.O.Box 1871, Mekelle, Ethiopia; 2 Department of Pharmacy, College of Medicine and Health Sciences, Adigrat University, P.O.Box 50, Adigrat, Ethiopia; 3 Student in School of Public Health, University of Saskatchewan, Saskatoon, SK Canada

**Keywords:** α-Amylase, *Aloe megalacantha*, *Aloe monticola*, Isolates

## Abstract

**Background:**

About 425 million adults had diabetes mellitus globally in 2017. Type 2 diabetes accounts for the enormous majority of diabetes cases and it is gradually growing which is predicted to increase by 48% in 2045. Imbalanced cellular carbohydrate and lipid metabolism cause an increase in postprandial blood glucose level which eventually leads to the onset and progression of type 2 diabetes mellitus. The lack of effective and safe carbohydrate hydrolyzing enzyme inhibitors contributes to the increasing prevalence. Thus, this study was targeted to assess the α-amylase inhibitory potential of isolates obtained from *Aloe megalacantha* Baker and *Aloe monticola* Reynolds, which are among the commonly used folkloric remedies for the management of diabetes mellitus.

**Method:**

The α-amylase inhibitory effect of *Aloe megalacantha* Baker and *Aloe monticola* Reynolds were evaluated using the 3,5-dinitro salicylic acid method. 2, 2-Diphenyl-2-picrylhydrazyl free radical scavenging property was also used to test the antioxidant effect of both plants. Results were analysed using GraphPad Prism software version 8.

**Results:**

The more polar isolates (AM_1_ and AG_1_) were possessed stronger α-amylase inhibition activity than the leaves latex and the other strains (AM_2_ and AG_2_). Leaf latex of *A. megalacantha*, AM_1_, AM_2_**,** leaf latex of *A. monticola*, AG_1_, and AG_2_ were found to have an IC_50_ value of 74.76 ± 1.98, 37.83 ± 3.31, 96.75 ± 1.98, 78.10 ± 1.88, 56.95 ± 1.88 and 64.03 ± 3.60 μg/mL, respectively (*P < 0.001*). The leaf latexes of *A. megalacantha* and *A. monticola* showed a significant (*P < 0.001*) free radical hunting property with an IC_50_ value of 890.1 ± 1.73 and 597.5 ± 2.02 μg/mL, respectively.

**Conclusion:**

Hence, the outcomes of the present investigation partly justify the acclaimed use of *Aloe megalacantha* and *Aloe monticola* for the treatment of diabetes.

## Background

Diabetes mellitus is among the chronic illnesses that develops either when there is insulin deficiency or when the body uses insulin ineffectively [1]. It has been estimated that 8.8% of the adult populations aged 20–79 years had diabetes mellitus in 2017 and it is projected to be 9.9% of the adult populations in 2045**.** It has been estimated that approximately five million deaths worldwide were due to diabetes mellitus in 2017 [2]. Beyond 90% of the cases are type 2 diabetes and the patients have complications like cardiovascular diseases associated with the oxidative stress secondary to the release of free radicals [2, 3]. Furthermore, more than 500 million type 2 diabetes mellitus cases were estimated worldwide in 2018, and the prevalence is similar between developed and developing countries. The incidence of type 2 diabetes in 2028 is forecasted to increase globally, with the majority in low-income countries [4].

Even though genetic has a part in the pathogenesis of non-insulin dependent diabetes, an unhealthy diet and a sedentary lifestyle are also important determinants [5]. Unregulated carbohydrate and lipid metabolism is also highly associated with postprandial hyperglycemia and eventually causes non-insulin dependent type 2 diabetes mellitus. Thus, inhibiting the action of carbohydrate digesting enzymes like α-amylase and α-glycosidase is an alternative treatment modality for type 2 diabetes. However, the synthetic α-amylase and α-glycosidase inhibitors are causing severe gastrointestinal side effects [6, 7]. Consequently, scientists are looking for an alternative remedies to prevent and treat diabetes type-2. Folkloric medicinal plants which have an antihyperglycemic effect through the inhibition of these enzymes and their antioxidant property are an excellent alternative to treat type 2 diabetes mellitus conditions and complications [8].

Different communities in Africa use *Aloe* plants for the management of diabetes mellitus, malaria, gastrointestinal problems, pain, inflammation, and snakebite [9–13]. There are approximately 600 different globally identified *Aloe* species; of these 46 species are native to Ethiopia [14–16]. Leaf latexes of *Aloe megalacantha* Baker and *Aloe monticola* Reynolds are among the most commonly used traditional medicines by the residents of northern and eastern Ethiopia. They use to take a coffee cup of fresh leaf latexes every morning for a month and sometimes longer for the management of diabetes mellitus [12, Personal communication].

The leaf latex of *A. megalacantha* was found to possess a significant antimalarial effect [17], wound healing activity, and good anti-inflammatory effect in mice models [18]. Thin layer chromatography (TLC) isolates gained from *A. megalacantha* leaf latex were also observed to possess promising antimalarial activity against *Plasmodium berghei* in mice model [17]. Isolated compounds gained from ethyl acetate extract of *A. megalacantha* root have exhibited good cytotoxic activity as compared with cryptophycin-52 and griseofulvin on human cervix carcinoma cell line KB-3-1 [19].

Leaf latex of *A. megalacantha* was reported to exhibit significant anti-hyperglycemic property in streptozotocin-induced diabetic mice [20], though the latex is not investigated for its potential in vitro α-amylase inhibition property. Interestingly, the responsible isolates for the antidiabetic activity of the latex were not tested for their in vivo antidiabetic activity. Plant-derived α-amylase inhibitors are important alternative remedies to treat type 2 diabetes by reducing the risk of postprandial hyperglycemia [6, 7]. Hence, this investigation was directed to evaluate the α-amylase inhibition property of TLC isolates obtained from the leaves latexes of *A. megalacantha* and *A. monticola* in order to in part propose the possible mechanism of their antidiabetic activities.

## Methods

### Plant materials collection

The leaves latexes of *A. megalacantha* and *A. monticola* were collected from the Tigray floristic region located north of the capital city, Addis Ababa, Ethiopia. Professor Sebsebe Demissew authenticated the plant material and the specimen was deposited with a voucher number 001 and 002 in National Herbarium, Addis Ababa University.

### Chemicals, reagents, and equipment

Chloroform, methanol (Calro Ebra reagents, France), type G silica gel F_254_ (Sigma-Aldrich, USA), 3,5-dinitro salicylic acid (DNSA) (Sigma-Aldrich, USA), 1,1-diphenyl-2-picrylhydrazyl radical (DPPH) (Sigma-Aldrich, USA) and α-amylase (HiMedia, Mumbai, India) were used to conduct this study, and all were analytical grade and procured from the market.

20  20 cm glass plate, thin-layer chromatography (TLC) jar (CAMAG, Germany), automatic TLC coating machine (CAMAG, Germany), ultraviolet (UV) lamp (CAMAG, Germany), double beam ultraviolet-visible (UV-VIS) spectrophotometer (P.G. instrument ltd., China), test tubes, micropipette and analytical weighing balance (adventurer OHAUS, China) were the equipments employed to perform this investigation

### Extract preparation

The leaf exudates of each plant were collected as per the method detailed by Hintsa et al. [17]. The leaves of both *Aloe* species were sliced transversally around their base and then the leaves were inclined towards the collecting bidet to gather the yellowish exudate of the leaves. At the end, the collected exudates were placed at the sheltered open air for 3 days by spreading on a plastic sheet until dryness which then revealed dried latexes.

### Preparation of isolates

Preparative thin layer chromatography (pTLC) made by coating a glass plate using silica gel at 500 μm thickness with the automatic TLC coater was employed to obtain the different isolates from the leaf latex of each plant. During the procedure, the latexes dissolved in methanol were applied directly as a thin band over one side of the pre-coated pTLC and the chromatograms were developed using chloroform and methanol mixture in an 80:20 ratio as a solvent system [17, 21, 22].

Besides the initial evaluation using the daylight, visualization of the chromatographic zones was performed using a UV lamp at 254 and 366 nm wavelengths. Subsequently, the chromatographic zones of each plant were coded according to the increasing order of their retention factor (*R*_*f*_) values. Every chromatographic zones were scratched off carefully and distinctly from the developed TLC plate and dissolved in an equivalent mixture of methanol and chloroform, filtered using filter paper and concentrated. The separated components of the leaves latexes are called TLC isolates in this study. The *R*_*f*_ value of each isolates were calculated using:

$$ Rf\ \mathrm{of}\ \mathrm{the}\ \mathrm{isolate}=\frac{\mathrm{distance}\ \mathrm{travelled}\ \mathrm{by}\ \mathrm{the}\ \mathrm{isolate}\ \mathrm{in}\ \mathrm{cm}}{\mathrm{distance}\ \mathrm{travelled}\ \mathrm{by}\ \mathrm{the}\ \mathrm{solvent}\ \mathrm{in}\ \mathrm{cm}\ \left(\mathrm{solvent}\ \mathrm{front}\right)} $$

### In vitro α-amylase inhibition property

The chromogenic DNSA method was followed to assess the α-amylase inhibition property of the latexes and TLC isolates [23]. The total reaction mixture containing 1400 μl of buffer (0.02 M sodium phosphate and 0.006 M sodium chloride at pH 6.9), 50 μl of α-amylase (2 units/mL) and test samples (the latexes or TLC isolates) at concentrations from 6.25 to 100 μg/mL was incubated for 10 min at 37 °C. Then after, 500 μl of 1% (w/v) starch solution dissolved in the aforesaid buffer was added to each tube and incubated again for 15 min at 37 °C. One-milliliter of DNSA reagent prepared by dissolving 12 g of sodium potassium tartrate tetrahydrate in 8.0 mL of 2 M sodium hydroxide and 20 mL of 0.096 M of DNSA solution was added to the reaction tube and boiled in a water bath for 10 min to terminate the reaction. Then, the reaction mixtures were cooled to ambient temperature and read its absorbance at 540 nm by UV-VIS spectrometer. The control with absolute activity of the enzyme (Abs _control_) was performed following the above procedure in the absence of the test samples. A blank assay using the test samples in their respective doses in the absence of α-amylase was also done to avoid the absorbance effect of the test samples. Acarbose was used as a positive control following the above protocol used for the test samples at similar concentrations. The inhibitory property shown by the test samples and the standard drug were compared with the negative control (100% enzyme activity) and expressed as percent α-amylase inhibition. The percent inhibition was determined with the equation given below:

$$ \%\upalpha -\mathrm{amylase}\ \mathrm{inhibition}=\frac{\mathrm{Abs}\ \mathrm{control}-\mathrm{Abs}\ \mathrm{test}\ \mathrm{sample}\ }{\mathrm{Abs}\ \mathrm{control}} \times 100 $$

The percent inhibition of the latexes and TLC isolates were plotted against their concentrations and then, the concentration of each of the test samples which inhibits 50% of the enzyme activity (IC_50_ values) was acquired from the plot.

### **DPPH** radical scavenging property

The antioxidant effect of leaves latexes of *A. megalacantha* and *A. monticola* were measured using the in vitro DPPH scavenging assay according to the protocol stated by Braca et al. [24]. Concisely, 30 μL of test samples solution dissolved in methanol at different concentrations (1000, 500, 250, 125 and 62.5 μg/mL) were prepared from the leaf latexes of each plant and subsequently transferred to a distinct test tubes containing three mL of a 0.004% (w/v) DPPH radicals dissolved in methanol in separate test tubes. After keeping the reaction solution in dark for half an hour at ambient temperature, the absorbance of each solution was read at 517 nm by a UV-VIS spectrophotometer. Vitamin C was used as a reference drug and examined under the same conditions.

A blank solution prepared from methanol and DPPH solution in the absence of test samples was measured for its absorbance after it was kept in the dark place. A triplicate sample measurement was done and averaged. Sample concentration providing 50% inhibition (IC_50_), the amount of sample needed to hunt half of the DPPH radicals, was computed from a concentration versus % inhibition graph of each sample.

Radical scavenging property of the test samples was described using percent inhibition and computed by the equation beneath:

$$ \%\mathrm{DPPH}\ \mathrm{inhibition}=\frac{\mathrm{Abs}\ \mathrm{control}-\mathrm{Abs}\ \mathrm{test}\ \mathrm{sample}\ }{\mathrm{Abs}\ \mathrm{control}}\times 100 $$

Where Abs _control_ is the absorbance of the blank control in the absence of the test sample and Abs _test sample_ is the absorbance of the solutions that contains test samples.

### Data analysis

Results were computed by using GraphPad Prism version 8 (GraphPad Software, San Diego, USA) and reported as a mean ± standard deviation (M ± SD). The mean of each test sample was statistically compared to each other using a one-way ANOVA followed by Tukey’s HSD post hoc test for multiple comparisons. The differences were regarded as significant at *P < 0.05*.

## Results

### Isolates prepared from *A. megalacantha* and *A. monticola*

Two yellowish amorphous solid isolates were obtained from the leaf latex of *A. megalacantha* with *R*_*f*_ values of 0.33 and 0.49 that was coded as AM_1_ and AM_2_, respectively. Yellow and dark brown amorphous solid isolates were also obtained from the leaf latex of *A. monticola* and coded as AG_1_ (*R*_*f*_, 0.27) and AG_2_ (*R*_*f*_, 0.61). The isolates seemed as bright yellow under daylight and UV radiation of 366 nm and they looked like dark spots at 254 nm wavelength.

### In vitro α-amylase inhibition test

**Fig. 1:**
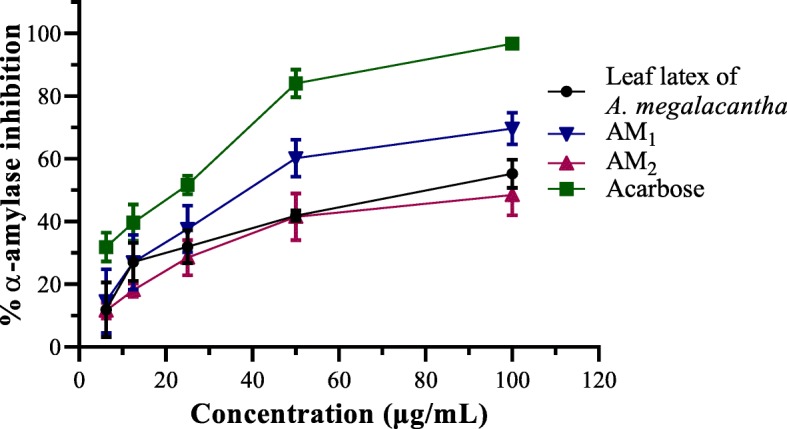
Plot of percent α-amylase inhibitory effect versus different concentrations of leaves latex, AM_1_, and AM_2_ of *A. megalacantha* Baker

**Fig. 2:**
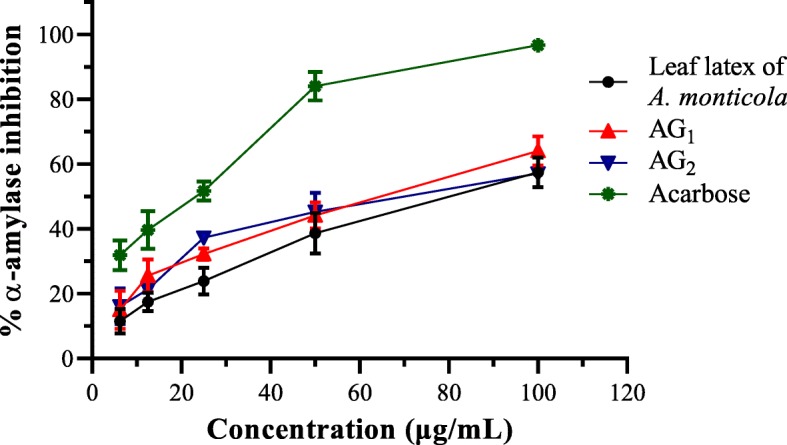
Plot of percent α-amylase inhibitory effect versus different concentrations of leaves latex, AG_1_, and AG_2_ of *A. monticola* Reynolds

Both the leaves latexes and TLC isolates of both plants were possessed important α-amylase suppression activity as presented in Figs. 1 and 2. All the test samples showed a dose-dependent α-amylase inhibitory effect with the most potent effect observed from AM_1_ and to some extent AG_1_.

**Fig. 3:**
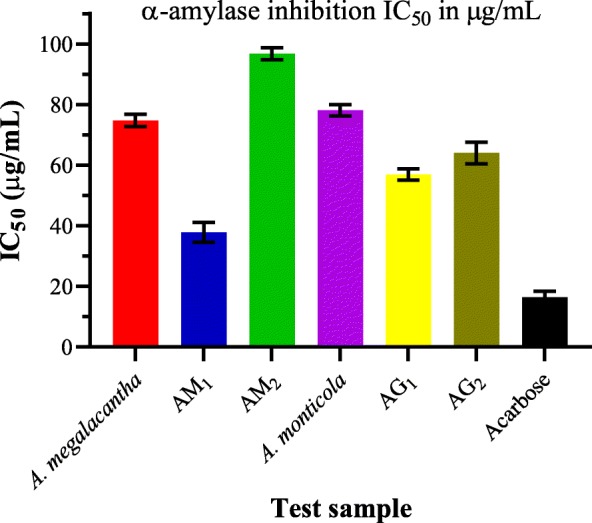
IC_50_ value of leaf latexes and TLC isolates of *A. megalacantha* and *A. monticola*

For the enzyme inhibition study, IC_50_ of all the test samples were determined from the graphs with AM_1_ exhibited the lowest IC_50_ of 37.83 ± 3.31 μg/mL when compared to the other test samples (Fig. 3). Leaf latex of *A. megalacantha*, AM_2_, leaf latex of *A. monticola*, AG_1_ and AG_2_ were also found to have an IC_50_ value of 74.76 ± 1.98, 96.75 ± 1.98, 78.10 ± 1.88, 56.95 ± 1.88 and 64.03 ± 3.60 μg/mL, respectively. Acarbose, the positive standard, showed an IC_50_ value of 16.49 ± 1.91 μg/mL. There was statistically significant difference (*P < 0.001*) between the IC_50_ values of the test samples and the standard drug. However, there was insignificant difference between the IC_50_ values of leaves latex of *A. megalacantha* and leaves latex of *A. monticola* (*P > 0.05*), and the isolates AG_1_ and AG_2_ had significantly different (*P < 0.05*) IC_50_ value.

### Antioxidant activity

**Fig. 4:**
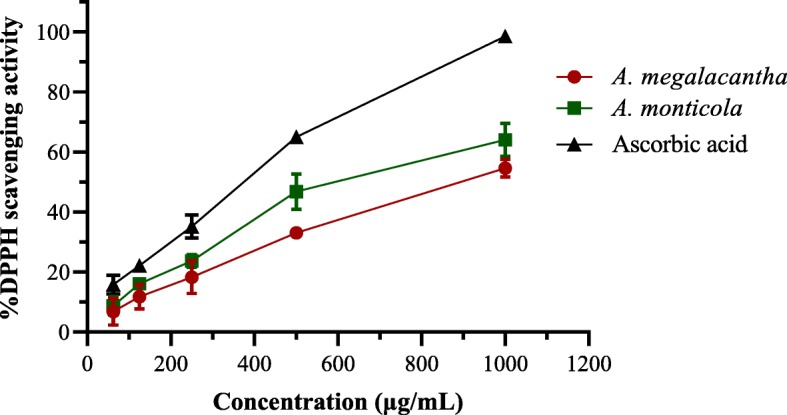
DPPH radical hunting property of the leaves latexes of *A. megalacantha* and *A. monticola* in comparison with the effect of ascorbic acid

The strength of radical scavenging properties of the leaves latexes were likened by calculating their amount that causes deduction of DPPH absorbance by half (IC_50_ value). As can be seen from Fig. 4, the leaf latexes of *A. megalacantha* and *A. monticola* have exhibited a prominent DPPH radical hunting property throughout the concentration ranges studied.

**Fig. 5:**
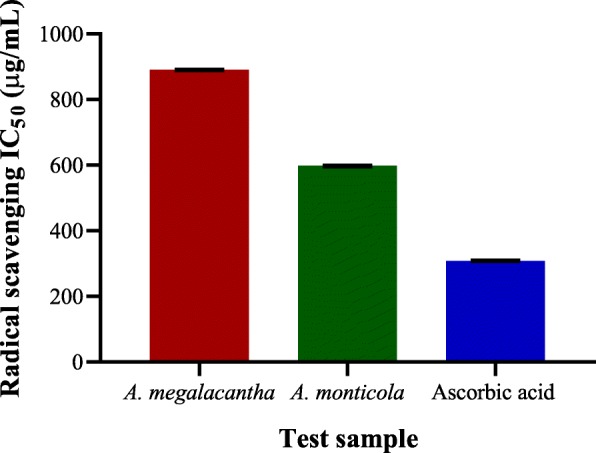
The IC_50_ values of leaves latexes of *A. megalacantha* and *A. monticola* against DPPH radical

The IC_50_ of the leaves latexes of *A. megalacantha* and *A. monticola* (Fig. 5) were calculated to be 890.1 ± 1.73 and 597.5 ± 2.02 μg/mL, respectively. The standard control (ascorbic acid) was observed to have a half inhibitory concentration of 308.4 ± 1.87 μg/mL. Both the leaf latexes have also possessed a noticeable DPPH radical hunting property compared to the blank control. The radical hunting IC_50_ values of the test samples were statistically significant (*P < 0.001*) as compared to each other.

## Discussion

AM_1_ and AG_1_, the TLC isolates separated from leaves latexes of *A. megalacantha* and *A. monticola* respectively*,* are considered to be more polar compounds than AM_2_ and AG_2_ as they have small *R*_*f*_ values during isolation using silica gel coated TLC plate with CHCl_3_:MeOH (80,20) solvent system. These isolates might also be glycosides of anthraquinones or its derivatives as they have similar retention factor with the previously isolated anthraquinone glycosides from leaf latex and root extracts of different *Aloe* species with the same method [9, 19, 21, 22, 25].

Lowering of postprandial blood glucose level by inhibiting the type 2 diabetes linked enzymes (α-amylase) was one alternative approach to treat type 2 diabetes. But severe gastrointestinal illnesses have been reported after oral administration of the currently available enzyme inhibitors such as miglitol and acarbose. Medicinal plant products which inhibit the action of α-amylase could lower the risk of hyperglycemia after food ingestion and may contribute in the management of non-insulin dependent diabetes mellitus [6, 7].

Even though there are limited in vitro antidiabetic studies conducted on *Aloe* plants, there are substantial reports regarding the in vivo antidiabetic activities of these plants. For instance, the high molecular mass portions of *A. vera*, which bears < 10 ppm of barbaloin, was associated with significant blood glucose-lowering capacity within six weeks in patients with diabetes mellitus. Prominent reduction in serum triglycerides was also noted after four weeks treatment with devoid of harmful effect on kidney and liver functions [26]. The previous study on the anti-diabetic activity of *A. vera* juice found that the oral dose of one teaspoon juice of *A. vera* in diabetic patients were observed to reduce blood sugar and triglyceride levels within two weeks [27]. The concurrent administration of *A. vera* juice and glibenclamide in diabetic patients caused significant reduction of blood sugar level after two weeks and blood triglycerides concentration after four weeks treatment with no toxic effects on kidney or liver function. However, glibenclamide alone didn’t cause a reduction in serum fasting glucose and triglycerides levels [28].

In another study, blood sugar concentrations of alloxan-induced hyperglycemic rats were reduced after the oral dose of the leaf extract of *A. vera*. The antidiabetic effect of this leaf extract at doses of 200 and 400 mg/kg was found equivalent to the reference drug metformin hydrochloride at 50 mg/kg [29]. Additionally, the powder obtained from *A. excelsa* [30] and latex of *A. megalacantha* leaves [20] brought about a dose-dependent and significant reduction of blood sugar concentrations of the streptozotocin-induced hyperglycemic rodents.

In the present investigation, leaf latexes and TLC isolates of both *A. megalacantha* and *A. monticola* were observed to possess statistically significant α-amylase inhibitory property in a concentration-dependent fashion. Both the leaves latexes of *A. megalacantha* and *A. monticola* were showed similar enzyme inhibition activity and that were better inhibitory effect than the effect exhibited by AM_2_ and AG_2_. The enzyme inhibitory IC_50_ value of the test samples were statistically significant (*P < 0.001*) as compared with the blank control. AM_1_ and AG_1_ had lower IC_50_ values than the other isolates and the leaf latexes, which were significant (*P < 0.001*) relative to the different test samples. Hence, the more polar isolates (AM_1_ and AG_1_) have exhibited higher α-amylase inhibitory effect than the less polar isolates (AM_2_ and AG_2_) which is consistent with the reported studies that higher α-amylase inhibition was observed from the aqueous extracts of both *A. vera* [31] and *Morinda lucida* [32].

Extracts of differently aged *A. vera* have been reported to exert significant antioxidant activity in DPPH radical hunting assay with the three-year-old *A. vera* showed higher radical scavenging activity when compared to α-tocopherol as well as the two- and four-year-old *A. vera* [33]. The extracts of *A. vera* gel and peel showed significant DPPH hunting property, 2,2′-azinobis(3-ethylbenzothiazoline-6-sulfonic acid) and iron-reducing antioxidant activity [34–36]. A study by Brhane et al. (2018) noted that the ethanolic gel extract of *A. adigratana* possessed a significant in-vitro superoxide radicals and nitric oxide scavenging activity [37].

The leaf latexes of both *A. megalacantha* and *A. monticola* have demonstrated a statistically significant (*P < 0.001*) antioxidant property against DPPH free radicals as compared to the blank solution and this effect is consistent with the previously reported antioxidant activity of different parts of *Aloe* plants. Hence, the significant DPPH radical scavenging effect of *A. megalacantha* and *A. monticola* leaf latexes may help to reduce the emergence of complications from diabetes mellitus in addition to their antidiabetic activity through α-amylase inhibition effect [8].

## Conclusion

The current investigation has demonstrated a significant and dose-dependent α-amylase inhibition activity of the leaves latexes and TLC isolates of both *A. megalacantha* and *A. monticola*. Besides, both the leaves latexes showed a significant DPPH radicals scavenging effect. The α-amylase blocking property with antioxidant activity of these plant products may help to reduce the post prandial blood sugar levels by which it could minimize the incidence and progression of complications. Thus, findings of the current study may in part support the traditional use of the leaf latexes of *A. megalacantha* and *A. monticola* for the management of non-insulin dependent diabetes mellitus. Further studies are indispensible to characterize the TLC isolates and to explore their in vivo antidiabetic activities.

## Data Availability

The data used and analysed in this study are available from the corresponding author on reasonable request.

## Abbreviations

ANOVA: Analysis of variance
DNSA: 3,5-dinitro salicylic acid
DPPH: 1,1-diphenyl-2-picrylhydrazyl
HSD: Honestly Significance Difference
IC_50_: Half inhibitory amount of a substance
pTLC: preparative Thin-Layer Chromatography
TLC: Thin-Layer Chromatography
UV-VIS: Ultraviolet-Visible
